# Book Reviews

**Published:** 1888-05

**Authors:** 


### BOOK-REVIEWS.

(I.) DISEASES OF THE SKIN. A MANUAL for Practitioners and Students. By W. Allan Jamieson, M. D., F. R. C. P. Ed., etc. Edinburgh: Young J. Pentland. Philadelphia: J. B. Lippincott Co. 1888.

(II.) DERMATITIS VENENATA. AN ACCOUNT of the Action of External Irritants upon the Skin. By James C. White, M. D., etc. Boston: Damrell & Upham. 1888.

(III.) A PRACTICAL TREATISE ON DISEASES of the Skin. By John V. Shoemaker, A. M., M. D., etc, New York: D. Appleton & Co. 1888.

(I.) The author of this volume has earned for himself a deserved reputation in the field of dermatological literature by his editorial supervision, for a number of years past, of the Periscope of Diseases of the Skin, in the now venerable and always valuable *Edinburgh Medical Journal*. He has also from time to time made contributions of note to general medical literature in connection with the same subject. He is a careful writer, a close observer, an indefatigable student, and easily second of the recognized Scottish writers in cutaneous medicine.

That he has the courage of his convictions is shown by the significant fact that in the twelve-month which has recorded the issue from the press of the large and comprehensive treatise on diseases of the skin written by his Scotch colleague, Dr. M'Call Anderson, of Glasgow, he has ventured to publish a work of similar scope. The present population of Edinburgh is less than 250,000, the town being in fact less populous than either San Francisco or Baltimore of American cities. It is, however, a town which has the unenviable notoriety of suffering from more than its share of cutaneous ailments, and in particular of surpassing in the frequency of occurrence of "the itch."

Dr. Jamieson's book is a clever and rather carefully written essay on the themes of which he treats, with certain defects which are only too apparent to the American reader. Among these may be mentioned, first, the conspicuous lack of a systematic arrangement of his several subjects, with separate chapters exclusively devoted to individual diseases, and the arrangement of the paragraphs devoted to each under the heads, pathology, etiology, etc.; second, the adoption of a scheme of classification devised by a single American writer, Dr. L. D. Bulkley, of New York, which has been adopted by no other author and no qualified body of men either at home or abroad; third, an acceptance of many of the doctrines proposed and sustained solely by Mr.

Jonathan Hutchinson, the eminent president of the Royal College of Surgeons in England.

This is not properly the occasion on which to discuss the special place which that distinguished gentleman has won for himself in the scientific pantheon. It is all that a large ambition might covet and a noble reputation fitly hold. As a surgeon, as an oculist, and as a dermatologist, he is equally expert, but it is to be laid to his charge that among living men there are few who have in their writings contributed more than he to that confusion in the mind of the average practitioner respecting diseases of the skin which has made dermatology the reproach of its sister sciences. His lack of system, his hasty enunciation of personal views which fail to receive support of observers of the same field in other countries when tested by the severe ordeal of extended personal experience, and his fondness for imitating the late Sir Erasmus Wilson in coining new phrases for the designation of well understood and properly classified phenomena, these are but a few of Mr. Hutchinson's scientific faults; and these are, it must be confessed, the lines in which Dr. Jamieson, when walking in the footsteps of the man he admires, has been occasionally diverted from the best course.

For example, our author, in discussing the subject of eczema, properly denies its contagiousness, but couples this with hints which look toward another possibility; for example, pointing out that an eczema sometimes spreads to regions whither its characteristic discharge has flowed. This is practically the doctrine of the old women in the nursery. It is true that an eczema occasionally extends to the parts whither its discharge has dropped down or flowed down; but it is also true not only that the patch may extend upward while the discharge is dropping downward over an unaffected and irresponsive integument, but also that the discharge is wholly incapable of acquiring any contagious quality in the only phases of eczema in which it is produced, viz., those of acute type, since it consists, for the larger part certainly, of the serum of the blood only, which traverses the swollen tissues with such rapidity as to be incapable of receiving from them any infective principle, were such present. If, as Jamieson states, it may by this means be "communicated to others in the same way," it is difficult to understand why the disease is not, at least, infectious, a fact denied by every writer of repute on the subject; and controverted by clinical experience the world over. The author, in this connection, credits Krock with the enunciation of the doctrine (in 1885), that new patches of disease on the skin of the eczematous patient are produced by a reflex along the trophic nerves, from the irritation set up in the patches of infiltration, where the disease originally appeared; but long before this date, Kaposi formally enunciated the same obvious truth in his excellent treatise.

The author devotes many pages to a consideration of the various features of eczema and other different disorders (*e. g.* pityriasis maculata et circinata, pityriasis rosea), under the title of "Lichen," a name which, as dissociated from lichen pilaris, lichen scrofulosorum, lichen planus, and lichen ruber, is slowly dying out of the confusion from which the immortal Hebra was early to remove it. It is almost incredible that a modern author should thus revive its shadowy and unsubstantial ghost. Jamieson also, in connection with the curious disorder named above (pityriasis maculata et circinata, pityriasis rosea, of Bazin), neglects to credit an American writer of Chicago, who, in the *British Medical Journal* of last year, was first to point out to Dr. Jamieson and his colleagues that Wilson had probably observed this disease when he wrote some of his descriptions of lichen circumscriptus and lichen marginatus.

The author has occasionally permitted himself to lapse into a carelessness of expression which is scarcely to be looked for in a formal treatise designed for students and practitioners. Though properly describing "crusts" among the lesions of the skin, he repeatedly employs as a substitute for this the word "scab," a term describing rather the indefinite ideas of the dispensary patient than the exacter conceptions of the dispensary physician. On page 411, we find, "should baldness have shown symptoms, more energetic measures *are* needed," etc. Again, p. 26, "inflammation is set up at the roots and beneath the nails."

It would appear that the author is not familiar with the American method of employing the annealed jeweler's broach for the purpose of removing hairs by electrolysis, as he commends for this purpose the far inferior "diamond" cambric needle, an instrument altogether too unyielding for the production of what a distinguished American teacher of the art has described as the "catheterization" of the hair-follicle.

On the whole, Dr. Jamieson's work may be viewed as a fair compend of the knowledge generally had on the subjects of which he treats, with the special advantage to Scotch readers of informing them of what Americans are doing and have done in the field covered.

We may possibly deplore the fact that a great deal of our chaff has thus been spread abroad over the world; but can note with some pride that the day which sees this work issue from the Edinburgh press, is not that of the past in which every contribution made to scientific literature on this side the Atlantic was despised in Great Britain. What has wrought the change? Is it an indefinite advance along the line, made by toilers and delvers in every department of science? This is the essential cause, but the major element of importance is the fact that right under the noses of the older writers in medicine, books by American authors are sold in Edinburgh, Glasgow, and London; studied by lay and professional readers alike; and are by no possibility any longer ignored by persons claiming to represent the best fruit of research in any department of medical science.

(II.) Professor White's essay on Dermatitis Medicamentosa, originally read as a paper before the American Dermatological Association, has grown into a book; and while it is not a large or a pretentious book, it is one of the very best kind offered to the library of the practitioner. For there are some books which contain facts good enough and valuable enough in themselves, but which may be found as well or more concisely set forth in another book or books upon the same shelf. Professor White's book is one of that precious order that contain facts nowhere so systematically explained as in its special pages. As a work of reference there is actually nothing to take its special and valuable place.

The action of external irritants upon the skin is so extensive and important, that its fullest exploration almost involves the study of human life from the moment it quits its sub-aqueous existence, to the hour when it ceases to respond to irritation of any kind. In a large sense, this might include all the diseases of the economy, and every source of variation from the standards of health of all the organs of the body. Professor White's treatise comprehends, however, more definitely the study of those dermatoses which originate in the direct action of externally applied irritants, whether of the animal, or vegetable, or mineral world, or of classes of matter less readily assigned to a special kingdom.

Few men are better fitted for such a task than our author. He is not only in reputation and position as a teacher the most venerable of American dermatologists, but is also an expert botanist, and one of the best comparative anatomists in the country. His knowledge is systematized to a degree rarely achieved by the most learned scientists of our day. He is, above all things, practical in the precepts which are suggested by his researches, and not without that cool disdain of ignorance and unsupported fact which is only equaled by his distrust of what is not demonstrable.

The chapters he has devoted to arsenical poisoning, resulting from the use of the metal in the ornamentation of articles for domestic purposes; on rhus poisoning; on the toxic effects of arnica when externally applied in certain cases, and on the bullous efflorescence occasionally encountered on the skins of newly-arrived immigrants, all exhibit the evidences of careful, original observation in connection with subjects long associated with Professor White's name and authority.

In the section devoted to the Solidago, or Golden-en Rod, the author describes a mild form of dermatitis which he has observed upon the hands of those who have handled the plant; but with characteristic caution, he hesitates to pronounce absolutely upon the cause in the case of those who, having been gathering wild-flowers in the woods, are there exposed unwittingly to the "ubiquitous rhus." But there are many reasons for believing that the suspicions of the author are more than well-founded. Not only is the plant irritating to the skin, but a number of asthmatic subjects of both sexes positively declare that accesses of dyspnœa are brought on both by direct exposure to the plant in contact and also, by breathing the air of the road-ways alongside of which the plant is flowering. The relation occasionally observed between asthma and eczema in individuals of susceptible skin and mucous membranes, illustrates the fact that these organs protest at times with equal energy against influences which, in the mass of men and women, produce only the faintest temporary impression.

Dr. White's valuable book is warmly commended to every practitioner who has an interest in accumulating a good medical library for reference purposes; as also to the dermatologist, the practical physician, the botanist, and the naturalist.

III. A great contrast is exhibited between Professor White's original work in the treatise just considered, and the volume written by Dr. Shoemaker, with regard to which the author says: "All that I claim in it as especially original is a statement of the relative effects and values of numerous agents tested in my own many years of clinical experience in the treatment of skin diseases."

It would be only just, on the basis of this plea, to admit fully the claim put forward for his statement. It presumably refers to the eight pages devoted to the oleates as topical applications in cutaneous disorders, the notoriety to say nothing of the value of several of which constitutes the author's chief claim upon the professional public for attention. Some of these special articles, such as the mercuric and lead oleates, had a value and usefulness which were recognized before Dr. Shoemaker's "many years of clinical experience." With these few exceptions, there will be difficulty in establishing the fact that the others have won for themselves any substantial place in dermatological therapeutics.

For what else is contained in the book, the author, making no special claim for originality, may be properly credited with making a fair compilation from the published works of other writers. He does not take the trouble to credit some of the gentlemen, who have written before, with what they have done. One might suppose that he would in his preface have acknowledged his indebtedness to the distinguished American author on dermatology, who is not only his professional colleague, but a resident of the same city in which Dr. Shoemaker resides. One could scarcely know from anything here set forth, that a considerable number of American dermatologists have been associated in the task of advancing the study of cutaneous medicine in this country by indefatigable work, have collated the only dermatological statistics worth consideration on this side of the Atlantic, and have contributed papers, edited journals, and written elaborate treatises in the special field which Dr. Shoemaker seeks to occupy for the purpose of "supplying the needs of the medical student and of the busy physician."

The result is that where the published text-books are defective, because not brought up to date, Dr. Shoemaker's pages, brought up to no later date, betray his failure to extend his studies into the fields where the later observers have been at work. For example, he devotes nine lines to a consideration of Duhring's "inflammatory fungoid neoplasm" under the head of sarcoma, exactly where the subject was left by Dr. Duhring when his last edition was issued from the press. But that distinguished author would scarcely think to-day, if revising the work he did so well in the past, of neglecting to separate all forms of sarcoma from the mycosis fungoides in whose lesions Auspitz and others have recognized micro-organisms. Even if not yet assuredly admitted to the separated and distinguished class of disorders where probably tuberculosis, lupus, syphilis, rhinoscleroma, and lepra have fixed themselves permanently, no writer can afford to-day to ignore the claim set forward to advance mycosis fungoides to this category. Yet the name does not even appear in Dr. Shoemaker's book. Nor, it may be mentioned in the same connection, could one gather from anything written in these pages, that there had even been a claim made that syphilis was entitled to such position; nor is it even hinted that a bacillus had ever strayed into the field of vision of a seeker for the essence of the potency of its venereal virus.

This is in truth the age of commercialism, in law, letters, society, and medicine. The work before us belongs to the commercial class of medical books. It contains nearly sixty pages of prescriptions for the relief of diseases of the skin. And while men and women believe that money is the chief end of man, perhaps they will continue to cling to the delusion which still smacks of the darkness of the middle ages, that a man can buy with money a prescription that will cure him of his skin disease. Why not "touch" again for the King's Evil, and hang for witchcraft?

AN INDEX OF MATERIA MEDICA, WITH PRESCRIPTION WRITING, INCLUDING PRACTICAL EXERCISES. By CHARLES H. MAY, M. D., and CHARLES F. MASON, M. D. Pp. vi and 267. New York: William Wood & Company. 1887. Chicago: W. T. Keener.

This is the last addition to Wood's Pocket Manuals. The authors have had large experience in coaching students for examination, and state in the preface that they think the book is what is needed by students preparing for examinations. The first part consists in a list of the officinal and many non-officinal drugs, with their source and dose. This list is alphabetically arranged, and has occasional blank pages for additions. The part of the book which treats of prescription writing is well done.

QUESTIONS AND ANSWERS ON THE ESSENTIALS OF PHYSIOLOGY, PREPARED ESPECIALLY FOR STUDENTS OF MEDICINE. By H. A. HARE, B. Sc., M. D. (Univ. of Pa.). Pp. vi and 170. Philadelphia: W. B. Saunders. 1888.

The author is well aware of the disfavor with which such books as this are received by the profession, and says in its defense that the short period of study required as preliminary for examinations for the medical degree makes such books necessary. The selections have been intelligently made; the style is clear. The book is not, in any sense, a substitute for one of the regular text-books upon the subject treated, but may be of value in preparing for examinations.

OBSTETRIC SYNOPSIS. By JOHN S. STEWART, M. D. Pp. x and 202. Small 12mo. Philadelphia: F. A. Davis. 1888.

The fundamental objection to all books of the class to which this belongs is that they attempt what is impossible. Satisfactory discussion of the subject cannot be accomplished in the space allowed, and as books of ready reference, they can hardly be more useful than the larger complete works. So far as it is possible under the conditions, the author has done his work well in this case. A cut and description of the new Stewart forceps is given. At a recent meeting of the Philadelphia Obstetrical Society, this forceps was not favorably received.

SIX HUNDRED MEDICAL DON'TS; OR, THE PHYSICIAN'S UTILITY ADVANCED. By F. C. VALENTINE, M. D. New York: G. W. Dillingham. 1888.

It appears from the title page that the author has also published, among other volumes, one on "Central American Medical Curiosities," and the average reader may fail to understand why this small one was not included in the larger work. However, the author has endeavored to write a book for the use of laymen, and one which he hopes will make them more reasonable and intelligent as patients. It contains 600 numbered paragraphs, each one of which begins with the word "Don't." The conception of the book is so fundamentally undignified, and its literary solecisms are so numerous, that it deserves emphatic adverse criticism.

ESSENTIALS OF CHEMISTRY AND TOXICOLOGY. FOR THE USE OF STUDENTS IN MEDICINE. By R. A. WITTHOUS, A. M., M. D. Second edition. 294 pages. Pocket size. New York: William Wood & Company.

This small work has already won a place in the library of the medical student, and in many instances it is the only work on chemistry with which he has any acquaintance.

Admitting that the use of such books is commendable, no exception can be taken to the present volume. It contains 1,053 questions, relating to those parts of chemistry chiefly interesting to the physician, and answers to the same, given in a clear and concise manner. Of books of its scope, it is undoubtedly among the best. But should the student be encouraged to use such manuals, which, of necessity, are almost devoid of one of the most valuable features of a scientific book, viz., systematic method, logically followed out?

The American medical student has his "essentials" of chemistry, anatomy, physiology, histology, and of half a dozen other things, but where, in all of these, does he find his scientific discipline?

A PRACTICAL TREATISE ON THE MEDICAL AND SURGICAL USES OF ELECTRICITY. By GEO. M. BEARD, A. M., M. D., and A. D. ROCKWELL, A. M., M. D. Sixth edition. Revised by A. D. Rockwell, M. D. 758 pages, with 196 illustrations. Large 8 vo. New York: Wm. Wood & Company. 1888. Chicago: W. T. Keener.

The first edition of this work was published seventeen years ago, and was, in many respects, a pioneer among books of this class. In that edition the authors refer to the many disadvantages under which they labored in the pursuit of their experimental investigations, and to the, perhaps, greater drawbacks which the reader would encounter in trying to follow them in the practical application of their results.

Although at that time several American instrument makers were furnishing electrical and other apparatus to the schools, very few were engaged in the manufacture of batteries and other electrical appliances suitable for the physician's practice. Besides this, few, if any, of the medical schools were giving a course in general chemistry and electricity sufficiently extended to make the successful application of electricity to medicine by the average medical man practically possible. Much of the apparatus used in their earlier work was devised by the authors themselves.

In presenting their book to the public, they divided into four sections—*Electro-Physics*, *Electro-Physiology*, *Electro-Therapeutics*, and *Electro-Surgery*.

This division is maintained in the later editions. Sections three and four are, of course, mainly practical, and give in detail the experience of the authors and others in the application of electricity to the treatment of a great variety of cases. The recent advances in the use of the current in gynæcology seem to be fully considered, as are also a large number of applications in the field of surgery. The illustrations and descriptions of new forms of apparatus are good. The first and second sections of the work are no less valuable than the two "practical" sections. These were written to supply the want of accurate information on the topics of Electro-Physics and Electro-Physiology in the medical literature of the time, and in the subsequent revisions they have been improved to keep abreast with later discoveries in those lines.

To understand and take advantage of all the possibilities of his batteries and coils, the physician must be conversant with a good many chapters of physics, as well as with physiology. The sections in question seem to cover the ground as fully as necessary. In the discussion of the measurement of strength of currents, a number of practical examples are given, which are certainly an aid to the beginner, and the statement of Ohm's law and its consequences, with application to electro-therapeutics, is scientifically accurate, yet easily understood.

On the whole, the work can be cordially recommended.

A MANUAL OF WEIGHTS AND MEASURES. By OSCAR OLDBERG, Ph. D. Second edition. Revised. Pages iii, 246. Chicago: W. T. Keener.

The first edition of this little work, issued three years ago, was, unfortunately, marred by a large number of typographical errors. These have been corrected fully in the present edition, to which, besides, some new matter has been added. As it now stands, the book possesses great practical value to pharmacists and physicians, as its information regarding weights and volumes of the various solutions employed in medicine is accurate and complete.

The origin and relations of the different systems of weights and measures employed by civilized nations is explained, and the discussion of the vexed question of the metric system in medicine is considered from the standpoint of a man thoroughly conversant with all its phases.

I. ATLAS OF VENEREAL AND SKIN DISEASES. Comprising original contributions and selections from the works of Professor M. KAPOSI, of Vienna, Dr. J. HUTCHINSON, of London, Professor J. NEUMANN, of Vienna, etc. Edited by PRINCE A. MORROW, A. M., M. D., Clinical Professor of Venereal Diseases, etc. New York: William Wood & Company. 1888. Fasciculi, i, ii, iii, iv.

II. CLINICAL ATLAS OF VENEREAL AND SKIN DISEASES. Including Diagnosis, Prognosis, and Treatment. By Professor ROBERT W. TAYLOR, M. D. To be issued in eight parts, aggregating fifty-eight large chromo lithograph plates, measuring fourteen by eighteen inches, and contaning about two hundred figures, many of them life-size, with selections from the works of Cullerier, Fox, Fournier, Hebra, etc. Philadelphia: Lea Brothers & Company. 1888.

The profession is to be congratulated upon the fact that the field of illustration of cutaneous and venereal diseases, so long neglected in this country, is at last well occupied by two men having the distinguished ability and reputation of Professors Taylor and Morrow. The advanced plates furnished, of each of these atlases, are equally good, both in execution and in fidelity to nature; and are of an excellence so nearly attaining the same standard that it would be invidious to make distinctions between them. Both authors have a large clinical experience, and both, when selecting subjects for illustration outside of that experience, have, for the most part, turned to the same excellent sources, viz., the published plates of such eminent dermatologists and syphilographers as Hutchinson, Kaposi, Fournier, Neumann, and Ricord.

We can cordially recommend these admirable illustrations of disease to the general practitioner and student, as well as to the special investigator of the branches illustrated.

IRREGULARITIES OF THE TEETH, AND THEIR TREATMENT. By EUGENE S. TALBOT, M. D., D.D.S., Professor of Dental Surgery in the Woman's Medical College, and Lecturer on Dental Pathology and Surgery in Rush Medical College, Chicago. With 152 illustrations. Octavo, pp. 163. Philadelphia: P. Blakiston, Son & Co. 1888.

This book is an epitome of the latest and most advanced ideas in relation to the causes and treatment of irregularities of the teeth, with illustrations of the appliances invented by the author and those of Drs. Patrick, Farrar, Coffin, Guilford, Byrnes, Kingsley, Allan, Richardson, Matteson, Magill and others. It contains 163 pages and 152 illustrations. Many of the latter are entirely new, the greater number of which belong to the peculiarities of arrangement of the teeth.

The author has divided his book into two parts; the first being devoted to a description of the anatomy of the maxillæ and the teeth and their normal relationship; the etiology of dental irregularities, acquired, hereditary and accidental; and a brief consideration of the causes of irregularities of the teeth in idiots.

The second deals with the treatment of the various forms of irregularities and the construction and adaptation of appliances for special as well as the more common cases, and will on this account be found of great value to both practitioners and students.

Dr. Talbot is to be congratulated upon the careful manner in which he has gathered together so many valuable principles and condensed them into so small a compass.

The book is well indexed and the publishers seem to have spared no pains in performing their part of the work. J. S. M.

